# Effect of 1,2-propanediol on the Critical Micelle Concentration of Decyltrimethylammonium Bromide at Temperatures from 293.15 K to 308.15 K

**DOI:** 10.3390/ijms232415884

**Published:** 2022-12-14

**Authors:** Carmen M. Romero, Andrea P. Escamilla, Ana C. F. Ribeiro, Miguel A. Esteso

**Affiliations:** 1 Departamento de Química, Universidad Nacional de Colombia, Bogotá 111311, Colombia; 2 Centro de Química de Coimbra, Department of Chemistry, University of Coimbra, 3004-535 Coimbra, Portugal; 3 Faculty of Health Sciences, Universidad Católica de Ávila, Calle Los Canteros s/n, 05005 Ávila, Spain; 4 U.D. Química Física, Universidad de Alcalá, 28805 Alcalá de Henares, Spain

**Keywords:** decyltrimethylammonium bromide, 1,2 propanediol, critical micellar concentration, surface tension, sound velocity, adiabatic compressibility

## Abstract

It is well known that polar organic compounds, such as alcohols and polyols, exert an appreciable influence on water structure and thus have important effects on surfactant micellization. These substances are often used to modify the properties of surfactants in aqueous solutions, increasing the practical applications they have in diverse industries. In this work, the critical micelle concentration (CMC) of decyltrimethylammonium bromide (C_10_TAB) in water and in 1,2-propanediol aqueous solutions was determined from both sound velocity and surface tension measurements as a function of surfactant concentration in the temperature range of (293.15 to 308.15) K. The critical micelle concentration of the surfactant increases as the concentration of 1,2-propanediol becomes higher, while the effect on temperature does not show important changes within the range considered. At the selected temperatures, the standard thermodynamic parameters of micellization suggests that the addition of 1,2-propanediol makes the micellization process less favorable. Thermodynamic analysis suggests that the micelle formation of C_10_TAB is an entropy-driven process at the temperatures considered in this study.

## 1. Introduction

The micelle formation of surfactants in a solution is the consequence of the hydrophobic interaction between the hydrocarbon chains of the surfactant hydrated molecules and the hydrophilic interactions between the polar or ionic groups and water. Upon dissolution in the aqueous solvent, the surfactant molecules exist as monomers at a low concentration. As the concentration increases and once a certain concentration, known as the critical micelle concentration (CMC), is reached, the surfactant molecules tend to aggregate, forming micelles to minimize the exposure of the nonpolar surface area to water. This situation is observed in the abrupt change of the physical properties of the surfactant, such as electrical conductivity, fluorescence, sound velocity, and surface tension, among others, as a function of concentration [1,2,3,4,5]. 

Cationic surfactants are amphiphilic compounds that carry a positive charge, which plays an important role in their scientific, industrial, medical, and biotechnological applications. They are frequently employed as fabric softeners, antistatic agents, and anticaking agents for fertilizers, corrosion inhibitors, pigment dispersants, and emulsifiers, and they exhibit biological activity against microorganisms [1,2,5,6]. Cationic surfactants are more active than anionic and non-ionic surfactants. The cationic head of the surfactant provides affinity toward polyanions, cell membranes, and intracellular organelles, such as a mitochondrion, yielding the development of mitochondrion medicine [6]. Although they are poorly compatible with ionic surfactants, they have the advantage of having a high compatibility with non-ionic and amphoteric surfactants [1].

Alkyltrimethylammonium bromides are cationic surfactants consisting of one or more alkyl chains and a hydrophilic headgroup with a positive charge. A special advantage of ternary ammonium salts is that, because the length of the hydrocarbon chain can be changed, they can be modified so that the micelle size and aggregation can be controlled to obtain the physicochemical properties required for their different applications. Furthermore, in these salts, the electrical positive charge on the molecule is not affected by pH changes, so they share the majority of the properties and applications of cationic surfactants [1,5,6].

The micellization of these salts in water has been studied by different techniques, and the critical micelle concentration at 298.15 K has been reported by several authors [3,4,5,6,7,8,9,10,11,12]. The effect of temperature on the micellization process has also been studied; it has been observed that the change in the critical micelle concentration shows a complex behavior that depends on the structure of the surfactant [1,2,3,4,5,6,9,10,11,12,13,14,15,16].

For smaller alkylammonium bromides and chlorides, several authors have reported a decrease in CMC as the temperature increases until it reaches a characteristic minimum value, and then it begins to increase [11,12,13]. For salts with a longer hydrocarbon chain, such as tetradecyltrimethylammonium chloride and bromide and hexadecyltrimethylammonium bromide, a slight increase in CMC with temperature has been observed. Some authors indicate that CMC increases with the temperature in a wide temperature range [9,17]. However, the information is confusing and, in some cases, contradictory [9,11,12].

Knowledge of the effect of small model substances on the CMC of surfactants is very important for its theoretical and practical purposes. Several studies show that the addition of organic compounds, such as alcohols and polyols, to surfactant aqueous solutions can be used to modulate the surfactant’s properties and their micellar behavior, allowing them to be used for different applications. The effect of different organic additives depends on both the nature of the surfactant and the additive, and it affects the formation of aggregates, the critical micellar concentration, and the characteristics of the micelles formed. 

The effect of primary alcohols, diols, and polyols on the micellization behavior of alkyltrimethylammonium bromides in aqueous solutions, and on their critical micellar concentration (CMC) in particular, has been extensively studied. In addition to their practical interest, they can provide important information about solute–solute and solute–solvent interactions in aqueous solutions. The studies clearly show that the influence of these compounds depends on the chain length of the additive, the number of hydroxyl groups, the polarity of the solvent, and the temperature [12,13,14,15,16,17,18,19,20,21,22,23,24,25,26,27,28,29,30,31,32,33,34,35,36]. These results are consistent with previous studies which show that the behavior of the water–alcohol or polyol mixtures is a consequence of the number and position of hydroxyl groups and the hydrocarbon chain length because it changes the hydrophobic–hydrophilic balance [36,37,38]. As a result, the micellar behavior of surfactants has a clear dependence on the number and position of hydroxyl groups. It has been found that the effect of additional hydroxyl groups in alcohols increases the critical micelle concentration of surfactants and that CMC increases with diol concentration [20,21,22,23,24,25,26,27,28,29,30,31,32,33,34,35].

Decyltrimethylammonium bromide (C_10_TAB) forms conventional micelles and its aggregation behavior has been studied in water by several authors [3,4,5,6,7,8,9,10,11], as well as in the presence of alcohols [12,13,17]. However, the information available in the literature about the effect of diols on the aggregation characteristics of C_10_TAB is scarce, particularly regarding the effect of 1,2-propanediol. This effect is interesting as it increases the CMC of cationic surfactants as diol concentration increases [22], while in some cases with cationic surfactants, it exerts the opposite effect, decreasing the CMC as diol concentration increases [39]. 

In order to evaluate the impact of additional hydroxyl groups on the behavior of surfactants, and considering that the CMC can serve as a measure of micelle stability in a given state, the effect of 1,2-propanediol upon the micellization behavior of aqueous solutions of C_10_TAB is studied at several temperatures in this work. The surfactant was selected because it is considered the smallest of the alkyltrimethylammonium bromides [10], and no information has been found about the effect of 1,2-propanediol. The results are discussed in terms of the effect of the diol on the cationic surfactant.

## 2. Results

Table 1, Table 2, Table 3 and Table 4 summarize the experimental data for the density *ρ*, sound velocity *u*, and surface tension *σ* of C_10_TAB in water and the aqueous solutions of 1,2-propanediol obtained in this work. The properties were measured below and above the critical micelle concentration of salt in water to determine the effect of diol, as well as the temperature effect on the critical micelle concentration of the alkyltrimethylammonium bromide.

At all temperatures and for very dilute solutions, the acoustic and surface properties of the aqueous solutions of 1,2-propanediol follow the behavior described in the literature [36,37,40,41,42]. 

**Figure 1:**
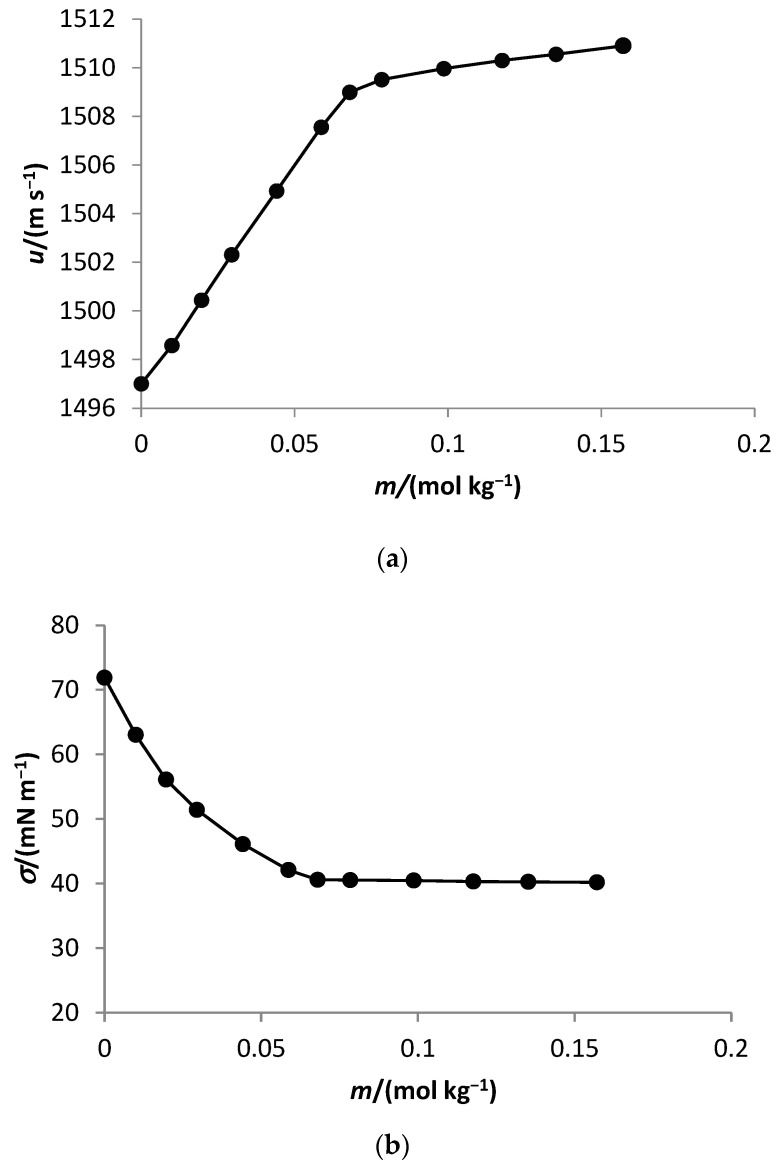
Sound velocity (**a**) and surface tension (**b**) of decyltrimethylammonium bromide in water as a function of molality at 298.15 K.

The behavior of the density, sound velocity, and surface tension at the selected temperatures of C_10_TAB in water follows the behavior described in the literature for the surfactants [3,4,5,6,7,8,9,10,11]. Figure 1 represents the behavior of the sound velocity and the surface tension of decyltrimethylammonium bromide in water as a function of molality at 298.15 K.

The same behavior of these properties was observed for the aqueous solutions of decyltrimethylammonium bromide in the presence of 1,2-propanediol. This experimental behavior follows the trend observed for solutions of alkylammonium bromide in the presence of alcohols [3,11,17,30].

The critical micelle concentration of C_10_TAB in water and aqueous solutions of 1,2-propanediol was determined from the dependence of sound speed and surface tension on surfactant concentration as these properties are sensitive to aggregation phenomena. A plot of the property as a function of salt concentration over a wide concentration range at each temperature exhibited two linear regions, corresponding to the premicellar region and the micellar region of the surfactant. 

The CMC values were found by plotting a graph of the sound velocity and surface tension as a function of the surfactant concentration. The abrupt change in the slopes of the two linear regions represented by the intersection of the two straight lines above and below the inflection point marks the CMC. The density increased with the surfactant concentration and without an abrupt change in the slope change. The results were used to calculate the surface tension of the solution.

Table 5 shows the CMC values obtained for the critical micelle concentration of C_10_TAB in water and aqueous solutions of 1,2-propanediol at temperatures between *T* = 293.15 K and 308.15 K obtained from sound velocity and surface tension measurements. 

The results obtained for the critical micelle concentration of C_10_TAB in water from both sound velocity and surface tension measurements are in good agreement. The results also present very good agreement with published values at 298.15 K [2,3,4,7,8,9,33,35].

The CMC of C_10_TAB is affected by the presence of 1,2-propanediol. The critical micelle concentration of C_10_TAB in the aqueous solutions of 1,2-propanediol exhibit a slight increase as the concentration of the diol increases. The change observed in the considered properties with the concentration follows the trend observed in other alkylammonium salts in aqueous solutions and in the presence of diols with a small hydrocarbon chain, such as ethylene glycol [16,18,19,20,21,22,23,24,25,26,27,28,29,30,31,32,33,34,35]. The change in CMC can be considered a consequence of the hydrophilic character of 1,2-propanediol that induces change in the surface of the micellar structure, making the aggregation process less favorable [31].

Some authors suggest that for ionic surfactants in water, the CMC exhibits a clear trend with the temperature, changing as the temperature increases and usually showing a concave curve and a characteristic minimum [44]. The CMC of C_10_TAB in water and aqueous solutions initially decreases as the temperature increases, reaching a minimum that becomes larger in the presence of propanediol and increases slightly with the diol concentration. This minimum temperature at which the micelle formation takes place is called the Krafft point or the Krafft temperature [11,38]. In this work, a similar dependence of the CMC on the surfactant in water with the relevant temperature was observed, as has been reported by other authors. The same trend was also observed in the presence of 1,2-propanediol. According to Zielinski [11] and Sengwa [38], the temperature minimum for C_10_TAB in water is located at around 300 K, which is very similar to the temperature at which the minimum was observed in this study. 

In the study presented by Boškovic et al., a similar effect of temperature on the CMC was observed for sodium dodecyl sulfate in water. In this study, the authors considered the effect of 1,2-propanediol on the CMC and on the thermodynamic parameters of the micellization of sodium dodecyl sulfate, and a different behavior was reported. The CMC decreases in the presence of 1,2-propanediol, but there is not a clear trend as the diol concentration increases [39].

Micellization is affected by the hydrophobic interaction between the hydrocarbon region of the surfactant molecules and the hydration and electrostatic interactions between the ionized headgroups with the surrounding counterions and water molecules. To obtain a complete description of the process of micelle formation, thermodynamic characterization is required, including the determination of the critical micelle concentration, the degree of micelle ionization, and the thermodynamics functions of micellization [12,13,31,43,44,45,46].

The thermodynamic parameters of micellization have been described by the models of mass action law and phase separation [12,43,44,45,46]. The standard Gibbs energy of micellization Δ_mic_*G*° was determined using the phase separation model. The following relation yields the calculation of the Gibbs energy for cationic surfactants [12,13,39,43,44,45,46,47]:

Δ_mic_*G*° = (2 − *β)RT* ln *X*_cmc_ (1)

where *X*_cmc_ is the mole fraction of the surfactant at the CMC, *β* is the degree of micelle ionization, *T* is the absolute temperature, and *R* is the universal gas constant. 

The degree of micelle ionization has been described by the pseudo-phase separation model, which considers free ions in the aqueous phase and bound ions in the micellar phase. The degree of micelle ionization plays an important role in micelle stability and in their growth. Its determination is essential to obtaining a complete thermodynamic description of micellar systems. Different experimental techniques can be used to measure the degree of micelle ionization. Among them, several techniques have been used, such as conductivity, surface tension, sound velocity, and viscosity, among others. However, the variability in *β* values using a particular technique and the variability observed for different techniques have been described. As Bales discussed, this is to be expected because each technique measures a different definition of ionic surfactant counterions [47].

Different researchers agree that the way to determine the degree of micelle ionization *β* is from the ratio of the slopes of the linear regions of the plot of the properties above and below the abrupt change of the slopes [12,13,16,33,44,45,46,47]. In this study, the value of *β* found for the surfactant in water was around 0.30, which is very similar to some of the values reported in the literature for this surfactant in water [13,33]. Considering that the dependence of CMC on temperature and diol concentration observed in our results is very small, it can be accepted that *β* is independent of temperature. On this basis, and because the temperature range used in this study is not too wide and the uncertainty in the degree of micellization is high, the ionization degree *β* was considered a constant, the value used being 0.30 [33,38]. Table 6 presents the degree of micelle ionization *β* and the standard thermodynamic parameters of micellization, calculated from sound velocity and surface tension measurements for C_10_TAB in water and aqueous solutions with different mole fractions of 1,2-propanediol at 298.15 K 

The standard enthalpy of micellization Δ_mic_*H*° was calculated from the following equation [13]:

(2) $$\Delta_{\mathrm{mic}}H{}^{\circ}=-{\mathrm{RT}}^{2}\;(2-\beta )\;{\left[\frac{\partial \ln X_{CMC}}{\partial T}\right]}_{P}$$

and the standard entropy of micellization Δ_mic_*S*° was obtained from the following equation:

Δ_mic_*G*° = Δ_mic_*H*° − *T*Δ_mic_*S*° (3)

Looking at the values summarized in Table 6, it can be seen that the values of the thermodynamic functions of micellization obtained in this work for C_10_TAB in water agree with those available in the literature [13,46,47]. The standard Gibbs energy of micellization is negative, indicating that the micellization process occurs spontaneously. On the other hand, because the temperature dependence of ln *X*_cmc_ is practically negligible and the temperature range studied is small, Δ_mic_*H*° is also very small and negative, indicating that the aggregation process is exothermic with a standard positive entropy of micellization of the surfactant. It has been observed that Δ_mic_*H*° for alkylammonium bromides with a chain length of *n*c 5 to *n*c 16 is negative and very small for salts with smaller chains, becoming positive as the chain length becomes larger. This has been considered a consequence of the predominance of the hydrophobic interaction 47]. Because the enthalpy value for C_10_TAB is close to zero, the enthalpic contribution to the micellization process can be considered very scarce while the entropy term −*T*Δ_mic_*S*° exerts the dominant effect in the micellization process and is responsible for the negative value of the standard Gibbs energy of micellization Δ_mic_*G*°.

As it can be ascertained from Table 6, the standard Gibbs energy decreases with the addition of 1,2 propanediol, and the values become less negative as the concentration of diol increases. Similarly, the standard enthalpy of micellization decreases with the diol concentration. The change observed indicates that the diol causes micelle destabilization, as it has been observed in other small diols. This behavior agrees with the trend followed by the CMC as the diol concentration increases and could be due to the hydrophilic character of 1,2-propanediol that affects the hydration layer of the micelle, making the aggregation process less favorable [20,21,22]. Because the enthalpy is so small, the standard entropy of micellization remains approximately constant as the diol concentration becomes larger. Thermodynamic analysis suggests that the micelle formation of C_10_TAB is an entropy-driven process at the temperatures considered in this study.

## 3. Conclusions

The effect of 1,2 propanediol on the physicochemical behavior of decyltrimethylammonium bromide (C_10_TAB) was investigated using sound velocity and surface tension measurements in the temperature range of (293.15 to 308.15) K.

The critical micelle concentration of C_10_TAB in aqueous solutions of 1,2-propanediol exhibited a slight increase as the concentration of the diol increased. This effect can be attributed to the hydrophilic character of 1,2-propanediol that induces changes in the surface of the micellar structure, making the aggregation process less favorable. Considering that the dependence of CMC on temperature and diol concentration observed in our results is very small, the degree of micelle ionization *β* was treated as a constant.

The values of the thermodynamic functions of the micellization of C_10_TAB in water and 1,2-propanediol aqueous solutions were determined using the pseudo-phase separation model from the temperature dependence of the CMC values. The values of Δ_mic_*G*° and Δ_mic_*H*° were negative and the values of Δ_mic_*S*° were positive, indicating that the hydrophilic character of 1,2-propanediol makes the aggregation process less favorable. Thermodynamic analysis suggested that the micelle formation of C_10_TAB is an entropy-driven process at the temperatures considered in this study.

## 4. Materials and Methods

Table 7 shows the characteristics of the reagents used according to the analysis certificates. Chemicals (C_10_TAB, Alfa Aesar, purity (mass fraction) 0.98 and 1,2-Propanediol, Alfa Aesar, purity (mass fraction) >0.99, Thermo Fisher Scientific Chemicals, Inc., Ward Hill, MA 01835-8099, USA) were used without further purification. The mass fraction purity is reported according to the certificates of analysis given by the suppliers. C_10_TAB was placed in a desiccator over silica gel for at least 72 h before use. Water was purified using a Barnstead Easy-Rodi D13321 system (Thermo Fisher Scientific, Marietta, OH 45750, USA) and degassed before use, employing a Cole Palmer 8895-25 ultrasonic cleaner (Cole-Parmer, Vernon Hills, IL 60061, USA) and obtaining water with a conductivity below 1.5 μS/m. All solutions were prepared by weight using a Mettler balance AT 201 (Marshall Scientific LLC, Hampton, NH 03842, USA) dual range with a readability of 1 × 10^−5^ g and a reproducibility better than 1 × 10^−5^ g in the lower range. In the preparation of the solutions and in the calculation of the standard uncertainty of molality, the purity of each of the reagents was taken into account.

Measurements of density, sound velocity, and surface tension in the temperature range of 293.15 to 308.15 K were performed at 5 K intervals. Measurements of density and sound velocity as a function of concentration at each temperature were carried out using a vibrating-tube-density and sound-velocity meter Anton Paar DSA-5000 (Anton Paar GmbH, Graz, Austria), operating at 3 MHz with temperature control better than ±0.005 K. The instrument was calibrated with dry air and purified water at 293.15 K according to the recommendations of the manufacturer. The values of the density and isentropic compressibility of water at each temperature used in calibration were taken from the literature [50,51]. 

Density and sound velocity were measured for the aqueous solutions of 1,2-propanediol used as solvent and for C_10_TAB in water and three 1,2-propanediol aqueous solutions. The reported data for density and sound velocity are the average of three independent measurements that were reproducible within ±1 × 10^−3^ kg m^−3^ and ±3 × 10^−2^ m s^−1^, respectively, with an uncertainty of 0.150 kg m^−3^ for density and 2 m s^−1^ for sound velocity [52].

The surface tension *γ* measurements were obtained at temperatures between (293.15 and 308.15) K using a LAUDA TVT-2 drop volume tensiometer (LAUDA-Brinkmann, Delran, NJ 08075, USA) with temperature control better than 0.1 K. The instrument is based on the principle of the pending-drop volume; the syringe used for the measurements had a volume of 1.0 cm^3^ and the inner radius of the capillary was 1.08 mm. The tensiometer was checked with pure water at 298.15 K, as recommended by the manufacturer, and at all the working temperatures by comparison with the literature [41]. The reported values are the average of 18 to 24 measurements, and the uncertainty in the surface tension measurements was ±10^−2^ mN m^−1^.

## Figures and Tables

**Table 1:** Density, sound velocity, and surface tension of decyltrimethylammonium bromide (C_10_TAB) in water at temperatures between T = 293.15 K and 308.15 K at 75 kPa.

*m*/(mol⋅kg^−1^)	*ρ*/(g⋅cm^−3^)	*u*/(m⋅s^−1^)	*σ*/(mN⋅m^−1^)	*ρ*/(g⋅cm^−3^)	*u*/(m⋅s^−1^)	*σ*/(mN⋅m^−1^)
293.15 K				298.15 K		
0	0.998203	1482.66	72.55	0.997047	1497.00	71.89
0.00998	0.998475	1484.78	63.35	0.997353	1498.58	63.04
0.01966	0.998733	1486.74	56.47	0.997592	1500.44	56.10
0.02952	0.998987	1488.78	51.55	0.997830	1502.31	51.42
0.04417	0.999347	1491.66	46.30	0.998170	1504.93	46.10
0.05870	0.999686	1494.61	42.17	0.998494	1507.55	42.10
0.06800	0.999892	1496.25	40.72	0.998694	1508.99	40.58
0.07841	1.000113	1496.81	40.54	0.998911	1509.51	40.56
0.09865	1.000516	1497.51	40.48	0.999312	1509.96	40.47
0.11767	1.000859	1497.97	40.28	0.999664	1510.30	40.31
0.13524	1.001147	1498.36	40.19	0.999970	1510.55	40.26
0.15712	1.001466	1498.81	40.06	1.000318	1510.90	40.24
303.15 K				308.15 K		
0	0.995645	1509.44	70.99	0.994029	1520.12	70.36
0.00998	0.995943	1510.82	62.88	0.994334	1521.33	62.70
0.01966	0.996173	1512.48	55.88	0.994556	1522.81	55.65
0.02952	0.996401	1514.16	51.28	0.994775	1524.36	51.14
0.04417	0.996728	1516.55	45.81	0.995090	1526.53	45.13
0.05870	0.997039	1518.92	42.08	0.995389	1528.65	42.05
0.06800	0.997230	1520.16	40.47	0.995573	1529.79	40.31
0.07841	0.997438	1520.63	40.39	0.995773	1530.19	40.18
0.09865	0.997822	1520.94	40.29	0.996142	1530.36	40.10
0.11767	0.998159	1521.15	40.14	0.996466	1530.45	40.06
0.13524	0.998450	1521.26	40.09	0.996744	1530.55	40.00
0.15712	0.998783	1521.50	40.05	0.997064	1530.68	39.95

Standard uncertainties are: *u*(*P*) = 1 kPa; *u*(*T*) = 0.01 K; *u*(*m* _C10TAB_) = 5 × 10^−5^ mol kg^−1^; *u(ρ) =* 0.150 kg m^−3^ (max); *u(u) =* 0.5 m s^−1^. Expanded uncertainty *U(σ) =* 0.12 mN m^−1^ (0.95 level of confidence).

**Table 2:** Density, sound velocity, and surface tension for decyltrimethylammonium bromide (C_10_TAB) in aqueous solutions of 1,2-propanediol X_OH_ = 0.02481 at temperatures between T = 293.15 K and 308.15 K at 75 kPa.

*m*/(mol⋅kg^−1^)	*ρ*/(g⋅cm^−3^)	*u*/(m⋅s^−1^)	*σ*/(mN⋅m^−1^)	*ρ*/(g⋅cm^−3^)	*u*/(m⋅s^−1^)	*σ*/(mN⋅m^−1^)
293.15 K				298.15 K		
0	1.00552	1544.89	61.39	1.004125	1553.96	60.79
0.00980	1.00579	1546.78	56.03	1.004380	1555.73	56.26
0.01934	1.00607	1548.29	51.50	1.004641	1556.97	51.45
0.02946	1.00635	1550.00	48.75	1.004903	1558.61	48.52
0.04412	1.00672	1552.63	44.33	1.005259	1560.89	44.15
0.05906	1.00707	1554.40	40.07	1.005590	1562.87	40.12
0.06864	1.00728	1555.65	39.64	1.005787	1563.67	39.58
0.07923	1.00749	1556.22	39.89	1.005989	1564.18	39.53
0.09779	1.00783	1556.15	39.58	1.006306	1564.00	39.49
0.11756	1.00813	1556.07	39.47	1.006592	1563.79	39.36
0.13768	1.00838	1555.96	39.32	1.006828	1563.60	39.30
0.15645	1.00856	1555.88	39.25	1.006998	1563.41	39.23
303.15 K				308.15 K		
0	1.002520	1561.82	60.51	1.000735	1568.42	59.92
0.00980	1.002763	1563.39	55.93	1.000945	1569.69	55.49
0.01934	1.003008	1564.48	51.34	1.001173	1570.64	51.10
0.02946	1.003256	1565.91	48.35	1.001403	1571.91	48.17
0.04412	1.003591	1567.95	44.10	1.001718	1573.69	44.00
0.05906	1.003904	1569.69	40.23	1.002014	1575.26	40.36
0.06864	1.004091	1570.42	39.62	1.002191	1575.82	39.49
0.07923	1.004283	1570.88	39.50	1.002374	1576.27	39.39
0.09779	1.004587	1570.65	39.36	1.002667	1575.97	39.32
0.11756	1.004862	1570.31	39.32	1.002936	1575.54	39.27
0.13768	1.005092	1570.10	39.20	1.003166	1575.10	39.15
0.15645	1.005260	1569.82	39.12	1.003341	1574.77	39.11

Standard uncertainties are: *u*(*P*) = 1 kPa; *u*(*T*) = 0.01 K; *u*(*m* _C10TAB_) = 5 × 10^−5^ mol kg^−1^; *u*(*x*_OH_) = 5 × 10^−5^; *u(ρ) =* 0.150 kg m^−3^ (max); *u(u) =* 0.5 m s^−1^. Expanded uncertainty *U(σ) =* 0.12 mN m^−1^ (0.95 level of confidence).

**Table 3:** Density, sound velocity, and surface tension for decyltrimethylammonium bromide (C_10_TAB) in aqueous solutions of 1,2-propanediol X_OH_ = 0.03466 at temperatures between *T* = 293.15 K and 308.15 K at 75 kPa.

*m*/(mol⋅kg^−1^)	*ρ*/(g⋅cm^−3^)	*u*/(m⋅s^−1^)	*σ*/(mN⋅m^−1^)	*ρ*/(g⋅cm^−3^)	*u*/(m⋅s^−1^)	*σ*/(mN⋅m^−1^)
293.15 K				298.15 K		
0	1.008555	1568.06	59.81	1.007058	1575.30	59.10
0.00961	1.008855	1569.45	55.44	1.007342	1576.54	55.25
0.01685	1.009040	1570.52	51.70	1.007519	1577.44	51.62
0.02938	1.009349	1572.48	48.17	1.007812	1579.28	48.11
0.04419	1.009693	1574.56	44.02	1.008139	1580.95	44.00
0.05896	1.010013	1576.37	40.95	1.008443	1582.54	40.97
0.06863	1.010211	1577.06	40.06	1.008631	1583.07	40.05
0.07851	1.010403	1577.77	39.73	1.008813	1583.66	39.71
0.08829	1.010583	1577.48	39.43	1.008984	1583.32	39.43
0.09819	1.010756	1577.26	39.28	1.009147	1583.04	39.15
0.11766	1.011065	1576.68	39.25	1.009440	1582.61	39.09
0.13600	1.011321	1576.34	39.13	1.009682	1582.22	38.98
0.15687	1.011571	1576.00	39.00	1.009916	1581.78	38.97
303.15 K				308.15 K		
0	1.005343	1581.28	58.42	1.003431	1586.09	57.85
0.00961	1.005623	1582.38	55.20	1.003612	1587.08	55.06
0.01685	1.005786	1583.20	51.45	1.003768	1587.99	51.30
0.02938	1.006057	1584.75	47.95	1.004026	1588.96	47.98
0.04419	1.006360	1586.34	43.99	1.004316	1590.36	43.98
0.05896	1.006644	1587.64	41.03	1.004587	1591.44	41.09
0.06863	1.006819	1588.11	40.09	1.004755	1591.85	40.14
0.07851	1.006991	1588.56	39.75	1.004919	1592.19	39.68
0.08829	1.007152	1588.31	39.46	1.005073	1591.87	39.41
0.09819	1.007308	1587.93	39.34	1.005222	1591.51	39.33
0.11766	1.007588	1587.45	39.13	1.005491	1590.95	39.11
0.13600	1.007824	1586.89	39.01	1.005717	1590.30	38.97
0.15687	1.008056	1586.27	38.94	1.005940	1589.72	38.83

Standard uncertainties are: *u*(*P*) = 1 kPa; *u*(*T*) = 0.01 K; *u*(*m* _C10TAB_) = 5 × 10^−5^ mol kg^−1^; *u*(*x*_OH_) = 5 × 10^−5^; *u(ρ) =* 0.150 kg m^−3^ (max); *u(u) =* 0.5 m s^−1^. Expanded uncertainty *U(σ) =* 0.12 mN m^−1^ (0.95 level of confidence).

**Table 4:** Density, sound velocity, and surface tension for decyltrimethylammonium bromide (C_10_TAB) in aqueous solutions of 1,2-propanediol X_OH_ = 0.04963 at temperatures between T = 293.15 K and 308.15 K at 75 kPa.

*m*/(mol⋅kg^−1^)	*ρ*/(g⋅cm^−3^)	*u*/(m⋅s^−1^)	*σ*/(mN⋅m^−1^)	*ρ*/(g⋅cm^−3^)	*u*/(m⋅s^−1^)	*σ*/(mN⋅m^−1^)
293.15 K				298.15 K		
0	1.012820	1599.25	57.02	1.011122	1603.79	56.28
0.00987	1.013052	1600.17	53.70	1.011337	1604.55	53.45
0.03011	1.013535	1602.50	47.06	1.011792	1606.50	47.03
0.04340	1.013828	1603.37	43.68	1.012069	1607.27	43.62
0.05884	1.014142	1604.92	40.75	1.012369	1608.46	40.80
0.06860	1.014328	1605.43	39.93	1.012546	1608.84	39.90
0.07863	1.014507	1605.79	39.57	1.012718	1609.30	39.54
0.08839	1.014671	1605.65	39.24	1.012876	1609.07	39.23
0.09846	1.014829	1605.33	39.14	1.013028	1608.67	39.06
0.11824	1.015106	1604.50	39.02	1.013299	1607.80	38.91
0.13769	1.015336	1603.86	38.84	1.013527	1607.10	38.80
0.14823	1.015443	1603.47	38.76	1.013635	1606.78	38.75
303.15 K				308.15 K		
0	1.009218	1607.02	55.71	1.007121	1609.30	54.99
0.00987	1.009423	1607.79	52.72	1.007308	1609.98	52.95
0.03011	1.009852	1609.45	46.50	1.007709	1611.29	46.97
0.04340	1.010113	1610.06	43.18	1.007953	1611.79	43.64
0.05884	1.010394	1611.07	40.54	1.008218	1612.57	41.01
0.06860	1.010560	1611.57	39.54	1.008374	1613.02	40.02
0.07863	1.010721	1611.73	39.08	1.008527	1613.16	39.51
0.08839	1.010869	1611.45	38.80	1.008668	1612.87	39.21
0.09846	1.011012	1610.98	38.60	1.008804	1612.34	39.03
0.11824	1.011265	1610.08	38.43	1.009047	1611.40	38.92
0.13769	1.011476	1609.28	38.32	1.009253	1610.52	38.77
0.14823	1.011576	1608.74	38.22	1.009351	1610.00	38.69

Standard uncertainties are: *u*(*P*) = 1 kPa; *u*(*T*) = 0.01 K; *u*(*m* _C10TAB_) = 5 × 10^−5^ mol kg^−1^; *u*(*x*_OH_) = 5 × 10^−5^; *u(ρ) =* 0.150 kg m^−3^ (max); *u(u) =* 0.5 m s^−1^. Expanded uncertainty *U(σ) =* 0.12 mN m^−1^ (0.95 level of confidence).

**Table 5:** Critical micelle concentration of decyltrimethylammonium bromide (C_10_TAB) in water and aqueous solutions of 1,2-propanediol at temperatures between T = 293.15 K and 308.15 K at 75 kPa from sound velocity and surface tension measurements.

*T*/K	10^2^⋅CMC_*u*_ /(mol⋅kg^−1^)	10^2^⋅CMC_σ_/(mol⋅kg^−1^)	10^2^⋅CMC_lit_/(mol⋅kg^−1^)
Water			
293.15	6.96	6.95	6.77 [11]
298.15	6.96	6.96	6.63 [11], 5.7 [7], 6.76 [9],
			6.0, 6.5 mol⋅L^−1^ [3],
			6.02 mol⋅L^−1^ [43]
303.15	6.92	6.93	6.50 [11]
308.15	6.96	6.97	6.68 [11]
X_OH_ = 0.02481			
293.15	7.06	7.06	
298.15	7.04	7.03	
303.15	7.04	7.03	
308.15	7.13	7.11	
X_OH_ = 0.03466			
293.15	7.12	7.13	
298.15	7.10	7.12	
303.15	7.10	7.10	
308.15	7.20	7.14	
X_OH_ = 0.04963			
293.15	7.33	7.33	
298.15	7.32	7.33	
303.15	7.32	7.32	
308.15	7.51	7.44	

Standard uncertainties are: *u*(*P*) = 1 kPa; *u*(*T*) = 0.01 K; *u*(*x*_OH_) = 5 × 10^−5^; *u*(CMC_u_) = 2 × 10^−4^ mol kg^−1^ (max); *u*(CMC_σ_) = 5 × 10^−4^ mol kg^−1^ (max).

**Table 6:** Standard values of the micellization thermodynamic parameters: Gibbs energy (Δ_mic_G°), enthalpy (Δ_mic_H°), and entropy (Δ_mic_S°) at the critical micelle mole fraction and degree of micelle ionization β of decyltrimethylammonium bromide (C_10_TAB) in water and in aqueous solutions with different mole fractions of 1,2-propanediol at 298.15 K and 75 kPa.

*X*_OH_	*β*	Δ_mic_*G*°/(kJ⋅mol^−1^)	Δ_mic_*H*°/(kJ⋅mol^−1^)	Δ_mic_*S*°/(J⋅mol^−1^K)
0	0.30	−28.15 ^a^; −29.0 ^b^; −29.2 ^c^	−0.10 ^a^; 0.00 ^b^; 0.2 ^c^	94.1 ^a^; 97.0 ^b^; 98.6 ^c^
0.02481	0.30	−28.12 ^a^	−0.03 ^a^	94.21 ^a^
0.03466	0.30	−28.07 ^a^	−0.03 ^a^	94.05 ^a^
0.04963	0.30	−27.94 ^a^	0.00 ^a^	93.71 ^a^

^a^ This work; ^b^ Ref. [48], ^c^ Ref. [49]. Standard uncertainties are: *u*(*P*) = 1 kPa; *u*(*T*) = 0.01 K; *u*(*x*_OH_) = 5 × 10^−5^; *u*(*β*) = 0.01. Relative standard uncertainties of the thermodynamic parameters are: *u*(Δ_mic_*G*°) = ±3%; *u*(Δ_mic_*H*°) = ±4%; *u*(Δ_mic_*S*°) = ±5%.

**Table 7:** Characteristics of the reagents.

Name	Source	CAS Number	Mass Fraction Purity
(C_10_TAB)	Alfa Aesar	2082-84-0	0.98
1,2-Propanediol	Alfa Aesar	57-55-6	>0.99

The mass fraction purity is reported according to the certificates of analysis given by the suppliers.

## Data Availability

Data are contained within the article.
